# Double Lip-An Atypical Facial Anomaly: Two Case Reports

**DOI:** 10.5005/jp-journals-10005-1556

**Published:** 2018-10-01

**Authors:** Namita Kalra, Rishi Tyagi, Amit Khatri, Amita Poswal, Gaurav Panwar, Kopal Garg

**Affiliations:** 1 Professor, Department of Pedodontics, University College of Medical Sciences and Guru Teg Bahadur Hospital, New Delhi, India; 2 Professor, Department of Pedodontics, University College of Medical Sciences and Guru Teg Bahadur Hospital, New Delhi, India; 3 Associate Professor, Department of Pedodontics, University College of Medical Sciences and Guru Teg Bahadur Hospital, New Delhi, India; 4 Senior Resident, Department of Pedodontics, University College of Medical Sciences and Guru Teg Bahadur Hospital, New Delhi, India; 5 Postgraduate Student, Department of Pedodontics, University College of Medical Sciences and Guru Teg Bahadur Hospital, New Delhi, India; 6 Postgraduate Student, Department of Pedodontics, University College of Medical Sciences and Guru Teg Bahadur Hospital, New Delhi, India

**Keywords:** Congenital, Cupid’s bow shaped, Double lip.

## Abstract

Double lip is a rare abnormality. It affects the lips, more often the upper lips and could be acquired or congenital. It may be associated with Ascher’s syndrome or occur in isolation. In this deformity, there is an accessory fold of redundant mucous membrane inside the vermillion border. This cupid’s bow-shaped accessory tissue is usually conspicuous during smiling but maybe occasionally visible even at rest. For the patient, this atypical facial deformity most importantly creates an aesthetic problem.

Nonetheless, it may also interfere with their speech or function. Surgical excision is the treatment of choice and gives appropriate esthetic and functional results. In this article, we have presented two case reports of congenital maxillary double lip. The etiology, clinical presentation, histopathology and treatment of this infrequent anomaly have been discussed.

**How to cite this article:** Kalra N, Tyagi R, Khatri A, Poswal A, Panwar G, Garg K. Double Lip-An Atypical Facial Anomaly: Two Case Reports. Int J Clin Pediatr Dent. 2018;11(5):451-455.

## INTRODUCTION

“Macrocheilia” or hamartoma^1^ commonly known as double lip, is an unusual abnormality which may be congenital or acquired. Although observed generally in the upper lip, cases affecting the lower lip and both upper and lower lips have been documented.^2^ This deformity is characterized by an accessory fold of redundant mucous membrane situated proximal to the vermillion border.

Double lip is typically discernible as two masses of hyperplastic tissue on both sides of the midline.^3,4^ Occasionally the hyperplastic tissues may not be symmetrical with one side being larger than the other.^5^ Infrequently a few cases of unilateral double lip have also been observed.^3^

Initially, it was established that double lip had no gender or race predilection.^2,6^ However, Palma and Taub in 2009 proposed that double more commonly affected the males in the ratio of 7:1.^5^

Clinically, a double lip is more obvious when the patient smiles or talks. While smiling, bilateral double lip typically shows a cupid’s bow appearance due to the retracted lip and the mucosa being positioned over the maxillary teeth.^7^

Surgical excision is the treatment of choice and is more often desired by the patients to correct their facial deformity. Nonetheless, the treatment could also be motivated due to interference during speech and mastication. Following surgery, recurrences are very rare, especially in congenital cases.^8,9^ Essentially the surgical excision should be limited to the mucosal and submucosal tissue with no involvement of the underlying muscle.^10,11^ Recurrences after surgery is rare.

The present article reports a series of two cases of congenital maxillary bilateral double lip.

## CASE REPORT

### Case 1

A male patient aged 8 years reported to the Outpatient Department of Pedodontics and Preventive Dentistry, The University College of Medical Sciences (UCMS) and Guru Teg Bahadur (GTB) hospital with the chief complaint of an unusually large and unappealing upper lip because of which the patient was ridiculed by his peers and relatives. The patient’s parents first noticed the abnormality 3 to 4 years back and reported that it insidiously enlarged over the years. The patient had no functional problems but wanted it corrected only for esthetic reasons. There was no previous history of trauma, any oral habits, infection or surgery on the lip. Presence of similar condition in any other family member or sibling was denied by his parents. Medical history of the patient was also non-contributory.

**Fig. 1:**
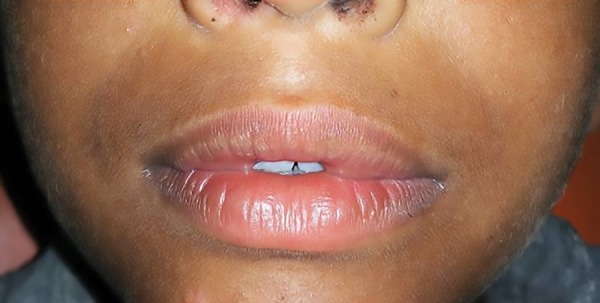
Preoperative picture of the patient when lips are at rest

**Fig. 2:**
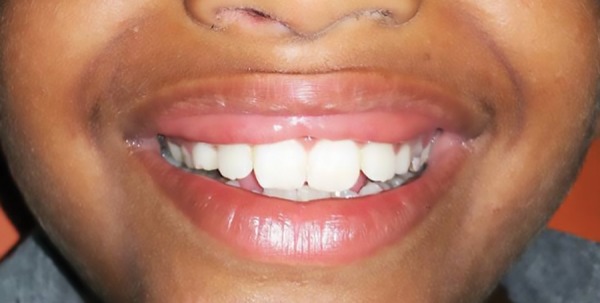
Preoperative picture showing Cupid’s bow shaped redundant tissue on smiling

On examination, an additional fold of bilateral mucosal tissue with a central constriction was observed on the inner aspect of the upper lip. Superficially, the mucosal surface was intact with no surface changes visible or palpable. The excess tissue was not very conspicuous when the patient’s lips were at rest (Fig. 1), but became very prominent when the patient smiled or spoke (Fig. 2). The congenital maxillary double lip was the provisional diagnosis made. There were no associated congenital oral defects. Blepharochalasis and thyroid enlargement as seen in Ascher’s syndrome were also absent. Blood profile was within normal limits and ultrasonography neck confirmed no thyroid enlargement. Hence surgical excision of the double lip under local anesthesia was planned.

**Fig. 3:**
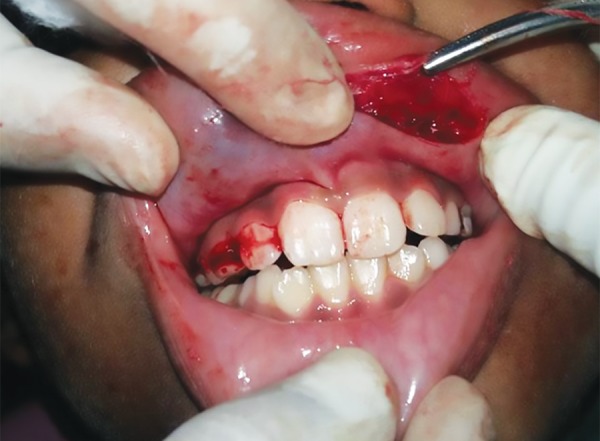
Intraoperative picture

**Fig. 4:**
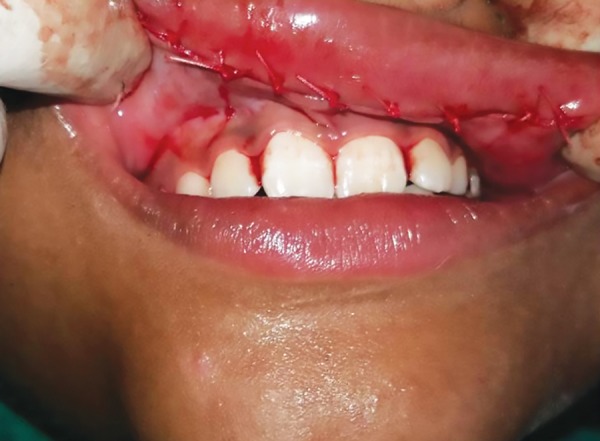
3-0 vicryl sutures placed after excision of redundant tissue

**Fig. 5:**
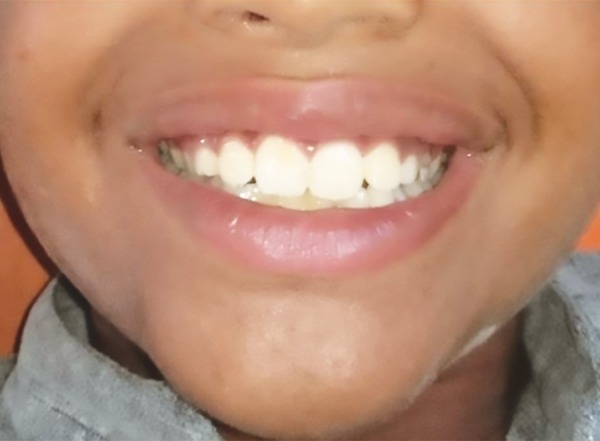
One-month postoperative picture

The redundant mucosal tissue was marked and local anesthesia administered. A transverse elliptical incision was given to excise the excess tissue (Fig. 3). Lip being highly vascular structure, local infiltration was chosen as the method of administering anesthesia because along with providing adequate anesthesia it also helped in obtaining hemostasis for a clean surgical field. The surgery was uneventful, and hemostasis was achieved by pressure packs. The hyperplastic mucosal tissue along with minor salivary glands present within this tissue was excised. Simple interrupted 3-0 vicryl sutures were given (Fig. 4) and light pressure pack placed over the upper lip for 24 hours. No postoperative complication was observed, and the results were very aesthetic. The patient was recalled after 24 hours, and then after 1 week for suture removal. The excised specimen was sent for biopsy and histopathology showed epithelium with underlying minor salivary glands. He was recalled after 1 month and kept on further follow-up. The parents and the child were both very satisfied with the results (Fig. 5).

### Case 2

A male patient of age 15 years reported to our department with the chief complaint of an abnormal and unsightly overgrowth beneath his upper lip because of which the patient was embarrassed and hence wanted to get it treated. The patient reported that though this overgrowth was present since birth, it gradually increased in size and the patient got extremely conscious about it at the age of 12 to 13 years. None of his family members presented with any such abnormality, and there was no history of any oral habits, trauma or surgery on the lip. The patient was medically fit and had no other functional complaints because of the presence of this defect.

**Fig. 6:**
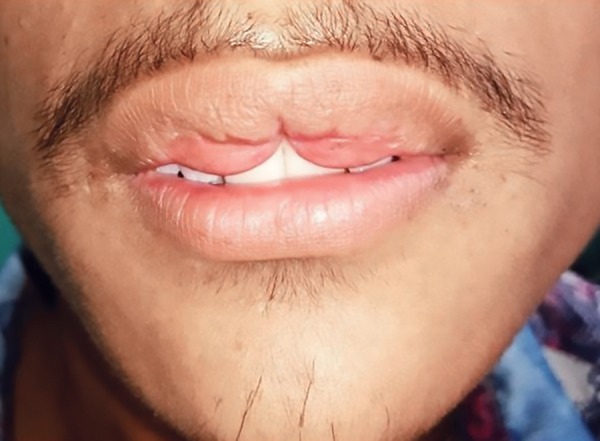
Deformity evident even at normal rest position of the lips

**Fig. 7:**
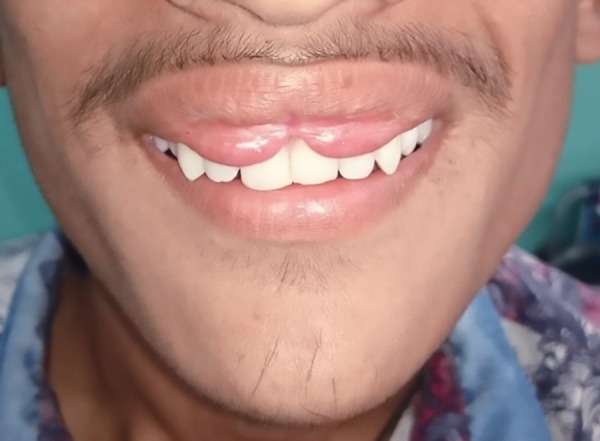
Increased prominence of the Cupid’s bow shaped accessory folds on smiling

Clinical examination revealed an extra fold of mucosal tissue bilaterally with midline constriction between the two mucosal bulges. The cupid’s bow-shaped redundant tissue was distinctively observable even when the lips were at rest (Fig. 6) and became even more prominent when the patient smiled (Fig. 7). It was an isolated lesion with no blepharochalasis, thyroid enlargement or any other associated congenital oral abnormality. A provisional diagnosis of the congenital double lip was made. Blood investigations were advised which revealed normal findings. Ultrasonography of the neck was done as a confirmatory diagnosis to rule out any nontoxic thyroid enlargement which together with double lip is a typical feature of Ascher’s syndrome. Hence surgery for the removal of the redundant fold of mucosal tissue was planned under local anesthesia.

**Fig. 8:**
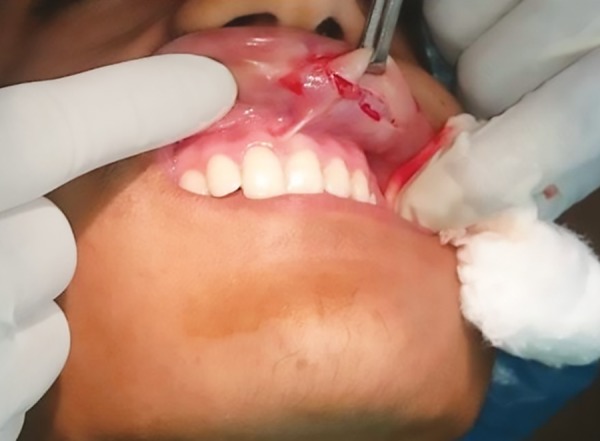
Intraoperative picture

**Fig. 9:**
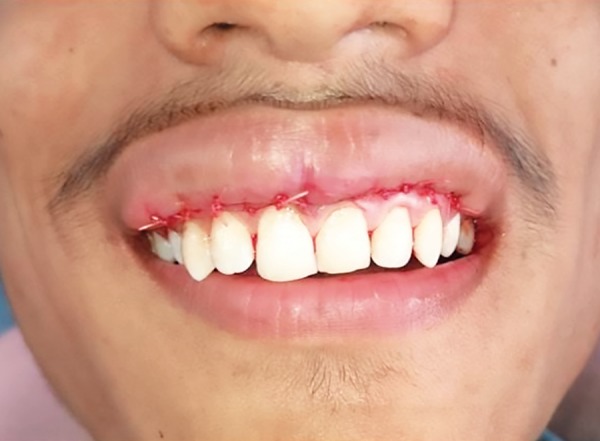
Immediate postoperative picture

**Fig. 10:**
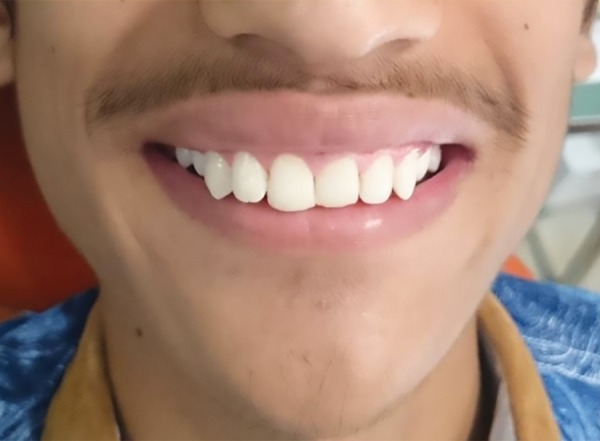
Picture of the patient after 1 month with excellent esthetic results

The surgical procedure was similar to the first case. The excessive mucosal folds were marked, local anesthesia administered by infiltration and tissue excised with the help of transverse elliptical incisions (Fig. 8). Careful attention was given that symmetrical tissue was excised on both sides. During the procedure, bleeding was not much and could be easily controlled by pressure packs. After excision, suturing was done to close the surgical defect by 3-0 Vicryl sutures (Fig. 9), and a light pressure pack over the upper lip was given for 24 hours. In this case, also no postoperative complications were seen, and the patient was very happy with his esthetics (Fig. 10). Histopathological findings of the excised specimen revealed mucosa with focal epithelial hyperplasia and underlying minor salivary glands. The patient was recalled after one week and sutures removed. He was evaluated after 1 month and kept on further follow-up.

## DISCUSSION

Double lip, though an extremely infrequent lip abnormality, when present causes significant difficulties for the patient.^5^ It is commonly observed that double lip is not quite conspicuous when the lips are at rest but the excess fold of tissue projects beyond the vermilion border when the lip is retracted as during smiling, laughing or talking.^12^ The above finding holds true for our first patient who was 8 years of age. However, in the second patient aged 15 years, the additional tissue was noticeable even at rest and became accentuated when the lips were in function. Thus, the above reaffirms the observation reported by various other authors that this defect possibly present since birth becomes more conspicuous as the patient grows.^4^

It has been suggested that with increasing age and eruption of teeth, double lip becomes more prominent, probably because of the repetitive “sucking-in” of this tissue in between teeth or malocclusion dentures.^13^

Coming to the etiology of the double lip, it may be congenital or acquired. The acquired form of the double lip may be secondary to trauma^14,15^ or oral habits such as sucking the lip between diastema^3^ or between ill-fitting dentures.^3,16^ The congenital type is a developmental anomaly.^14^ Both the cases in this article presented with a congenital form of the double lip. During fetal development, the tissue of the upper lip is divided into two transverse zones, a smooth outer zone closer to the skin (pars glabrosa) and a villous inner zone (pars villosa).^14^ Double lip has been proposed to arise by the hypertrophy of the pars villosa, during the second and third month of gestation because of the persistence of an exaggerated horizontal sulcus between the pars glabrosa and pars villosa of the developing lips.^3,9^

The double lip may be seen as an isolated lesion, or along with blepharochalasis and nontoxic thyroid enlargement. The triad of the above three lesions is a feature of Ascher’s syndrome (or Laffer-Ascher syndrome). Angioneurotic edema on both the eyelids as well as on the upper lip is prominent and diagnostic clinical symptoms of this syndrome.^17^ This syndrome was first described by Ascher, an ophthalmologist in 1920.^18^

A few cases of the double lip in association with other oral anomalies such as bifid uvula,^19^ hemangiomas,^20^ cleft palate, and cheilitis glandularis have also been documented.^2,20^

In both are cases, the double lip was present as an isolated lesion. It was not associated with any syndrome or any other oral anomaly.

Differential diagnosis of double lip could be a heman-gioma, lymphangioma, angioedema, cheilitis glandularis, cheilitis granulomatosa, mucous retention cyst, mucocele, salivary gland tumors, plasma cell cheilitis, and inflammatory fibrous hyperplasia.^3,4,20^

Nevertheless, Cupid’s bow appearance or midline constriction is a typical feature of the double lip and if present clearly distinguishes it from the other differentials.^21^

The treatment includes several surgical techniques like W-plasty,^21^ electrosurgical excision,^1^ and triangular excision.^10^ Transverse elliptical excision is used in most cases and gives good results.^19,22^ In both our cases we used transverse elliptical excision. Kenny^23^ had recommended bilateral infraorbital nerve blocks and mental nerve block to diminish the chance of tissue distortion when administering local anesthesia. Recurrence of this condition after surgical correction is extremely uncommon.^24^

## CONCLUSION

Double lip is a very rare abnormality, and its treatment is indicated chiefly due to its esthetic concern to the patient. Occasionally treatment could be motivated due to tissue interferes with mastication or speech or habits such as sucking or biting the extra fold of tissue.^3^ Recurrence of this condition after surgical correction is infrequent.

## CLINICAL SIGNIFICANCE

Double lip is of particular interest in dentistry because dentists are the first health professionals to identify and ascertain the diagnosis of this rare condition. A pediatric dentist will be an ideal candidate for early diagnosis and treatment of double lip, thereby reducing the psychological trauma to the patient because of its long-term presence.
